# Dr. Grissom’s Charges against Dr. Hammond

**Published:** 1878-12

**Authors:** 


### Dr. Grissom's Charges against Dr. Hammond.

Personality in journalism is to be reprobated, but it is possible for a physician to attain such eminence that his character is no longer a private but a public possession, the whole professihn being honored or dishonored with him. Such high station certainly has been reached by Prof. Hammond of New York, and as certainly serious attack upon his character is a matter not of private but of public moment. This much of apology we offer for noticing a present controversy, if the preferring and rebutting of criminal charges can be called a controversy. In a paper entitled "True and False Medical Experts," and more directly in subsequent writings, Dr. Grissom unskillfully, and with much of very objectionable matter, makes certain charges against the New York neurologist. Omitting the minor of these charges, the more serious may be stated succinctly as follows:

First. In order to clear McFarland, who shot Richardson, Dr. Hammond stated on the witness stand "that the insane are very persistent in their revenge. I have known insane men occupied with the idea of killing their keeper for years, and finally do it;" whilst in order to convict Montgomery he stated that "deliberation takes away the idea of an insane act."*

Second. That to insure the execution of Reynolds, Dr. Hammond declared under oath, "The disease [epileptic mania] is of remarkably short duration. There is not a case on record where it has lasted fifteen minutes;" whilst in order to convict Montgomery he had said, "when an epileptic has suffered from an attack, the mental disturbance continues, frequently, several days."

Third. That in the Johnston will case he gave testimony which was scientifically false, although necessary for the breaking of the will, and† that it was proven that he was to receive five hundred dollars for his testimony, and a contingent of twenty-five hundred dollars if he succeeded in breaking the will.

Fourth. That in the Montgomery case he one day gave one opinion and the following day an opposite one, having been seen in the mean while by the interested counsel.

Of course a wicked man may make a true accusation, and a man of good repute a false one. Nevertheless, the gravity of a charge

* We must refer to the pamphlets of Dr. Grissom to show how these various assertions were necessary to the escape or conviction of the accused.

†That there are no lucid intervals in monomania.

in a case like the present is enhanced by a previeus character for ability and probity on the part of the accuser. Dr. Grissom is a prominent alienist, of good repute among his associates, and he is a member of the *Judicial Council of the American Medical Association.* Under the circumstances it seems inconceivable but that he believes to be true what he asserts. To prefer such charges falsely, or even lightly, against a physician of Dr. Hammond's renown, would be professional suicide.

These considerations in no way prove the truth of the accusation, but they do remove the transaction from the arena of mere personal dispute and quarrel, and entitle it to rank as a semi-official citation of the asserted culprit before the profession, by one selected by the profession to judge of its ethical and moral questions. This view of the case is further strengthened by the somewhat defiant offer (in response to Dr. Hammond) of Dr. Grissom to prove his charges before a jury and to deposit sufficient bonds to cover any probable award or damage.

Under these circumstances the case assumes a most serious aspect. There is no medical man in these United States that could afford to allow his reputation to rest for a day in this position.

There is no medical body meeting in this country more reputable than the Association of the Superintendents of the American Institution for the insane. At the last meeting of this body a resolution was offered and warmly pressed by three members, condemning the action of Dr: Grissom. It was, however, defeated, we believe by an overwhelming majority. This is, of course, a virtual endorsement of the charges. So that, as the case now stands, the said charges have not only been publicly preferred against Dr. Hammond by a member of the Judicial Council of the American Medical Association, but have also been endorsed by the National Association of Specialists connected with the subject.

The reply to all this on the part of Dr. Hammond is to be found in two pamphlets, which may be analyzed as containing—first, various counter-charges against Dr. Grissom, with a flood of personal abuse; second, the assertion that the Association of Superintendents are endeavoring to destroy him (Dr. Hammond.) because he has been an advocate of the non-restraint system of treating insanity; third, the partial denial of some of the charges, but no straightforward, complete denial of them, and no attempt to disprove the detailed statements and documenttry evidence offered by Dr. Grissom.* A simple denial from Dr. Hammond of the accusations against him would bave deservedly had great weight with the profession; but his "open letters" are such a mixture of seeming evasion, school-boy wit, puerile abuse, and disgraceful

---

*It is but fair to state that the more serious of the counter-charges are disproven by documentary and other evidence by Dr. Grissom.

vulgarity* that we think they must have astonished his warmest friends. They certainly do not meet the needs of his cause at all. The profession will be very slow to believe his accusations against the Superintendents' Association, and it is he, Dr. Hammond, and not Dr. Grissom, who is at the bar of public opinion. If Dr. Hammond would retain any of the respect of the profession, he must make a brief and pointed but detailed denial of the charges, and follow this by citing Dr. Grissom either before the Judicial Council of the American Medical Association or before a jury in a libel suit. The professional tribunal is, to our thinking, the proper one; but if Dr. Hammond desires to recoup himself for the expense and annoyance to which, if innocent, he has been unnecessarily subjected, the civil court is open to him. Of one thing he may be certain,—that by his own replies he has done much to turn against himself the current of professional opinion, and that the circumstances of the case imperatively demand decisive action on his part.—*Editorial Philadelphia Medical Times.*

*To justify this assertion, we quote in full the latter part of the concluding paragraph of the second open letter. *Ex uno omnes disce.*

"A distempered and snarling cur has nobler mental and moral qualities than you; the vibrio that wriggles in decomposing filth is higher in the scale of existence; the foul bird that desecrates its own nest is less odious; the wretch who, actuated by perverted instincts, revels in nastiness and abominations, is not so execrable; the monster who insults the mother who bore him is more entitled to human sympathy. Whether you receive your dues in this world or in the next is, as I have said, a matter of no consequence to me; but if a being as groveling, hypocritical, fraudulent, and polluted as you, could through any means, escape punishment to which his crimes entitle him, then I should lose faith in the justice of an omniscient God, and wonder for what purpose hell was instituted. Go, graceless and irreclaimable quack; hardened and infamous blackguard, sane or insane, knave or maniac, and regale your devious fancies by the contemplation of your own depravity."

